# Involvement of the parabrachial nucleus in emergence from general anesthesia

**DOI:** 10.3389/fnins.2024.1500353

**Published:** 2024-12-11

**Authors:** Jia Li, Qiuyu Zhu, Jiaxin Xiang, Yiyong Wei, Donghang Zhang

**Affiliations:** ^1^Department of Anesthesiology, Honghui Hospital, Xi’an Jiaotong University, Xi’an, China; ^2^Department of Anesthesiology, The Second Affiliated Hospital, School of Medicine, The Chinese University of Hong Kong, Shenzhen & Longgang District People’s Hospital of Shenzhen, Shenzhen, China; ^3^Department of Anesthesiology, Weill Cornell Medicine, New York, NY, United States; ^4^Department of Anesthesiology, Longgang District Maternity & Child Healthcare Hospital of Shenzhen City, Longgang Maternity and Child Institute of Shantou University Medical College, Shenzhen, China; ^5^Department of Anesthesiology, West China Hospital, Sichuan University, Chengdu, China

**Keywords:** parabrachial nucleus, PBN, general anesthesia, general anesthetics, review

## Abstract

The parabrachial nucleus (PBN), located in the dorsolateral pons, is involved in many important biological functions, such as sensory signaling, feeding, defensive behaviors, fear, anxiety, and sleep–wake cycles. General anesthesia shares the classical feature of reversible loss of consciousness with natural sleep, and accumulating evidence has indicated that general anesthesia and sleep–wake behaviors share some common underlying neural mechanism. In recent years, emerging studies have investigated the involvement of PBN in emergence from general anesthesia, but divergence exists in terms of different types of general anesthetics or different durations of treatment with the same group of general anesthetics. Here, we reviewed the current literature and summarized the evidence about the contribution of PBN to general anesthesia.

## Introduction

The parabrachial nucleus (PBN) is located in the dorsolateral pons and is dissected into lateral and medial subregions by the superior cerebellar peduncle (Palmiter, 2018). The lateral and medial PBN further contain several subnuclei with different biological functions (Chiang et al., 2019). The PBN primarily receives inputs from the nucleus tractus solitarius (Roman et al., 2016), trigeminal spinal subnucleus caudalis (Nakaya et al., 2023), and dorsal horn of the spinal cord (Deng et al., 2020) and sends outputs widely to many brain regions, including the thalamus (Deng et al., 2020; Jarvie et al., 2021; Li et al., 2023), ventral tegmental area (VTA) (Zhang et al., 2021), basal forebrain (BF) (Qiu et al., 2016), hypothalamus (Benevento et al., 2024), insular cortex (Jarvie et al., 2021), periaqueductal gray (Chiang et al., 2020), bed nucleus of the stria terminalis (BNST) (Chiang et al., 2020), central amygdala (CeA) (Chiang et al., 2020), and reticular formation (Barik et al., 2018). The PBN is a well-known wake-promoting region (Qiu et al., 2016; Xing et al., 2024; Chen et al., 2023), and several projections involving the PBN, including the PBN-BF (Qiu et al., 2016; Xu et al., 2021), PBN-lateral hypothalamus (Qiu et al., 2016; Xu et al., 2021), and lateral hypothalamus-PBN (Wang et al., 2021), have been found to regulate sleep–wakefulness behaviors. In addition to regulating sleep–wakefulness behaviors, the PBN is also implicated in many other important physiological and pathological conditions, such as sensory sensation (Ren et al., 2023; Mu et al., 2017), defensive responses (Xie et al., 2022; Yang et al., 2023), feeding (Singh Alvarado et al., 2024), pain (Wu et al., 2023; Zhou et al., 2023), respiratory depression (Liu et al., 2021; Miller et al., 2017), and negative emotions (Wang et al., 2024; Chen et al., 2022).

**Figure 1:**
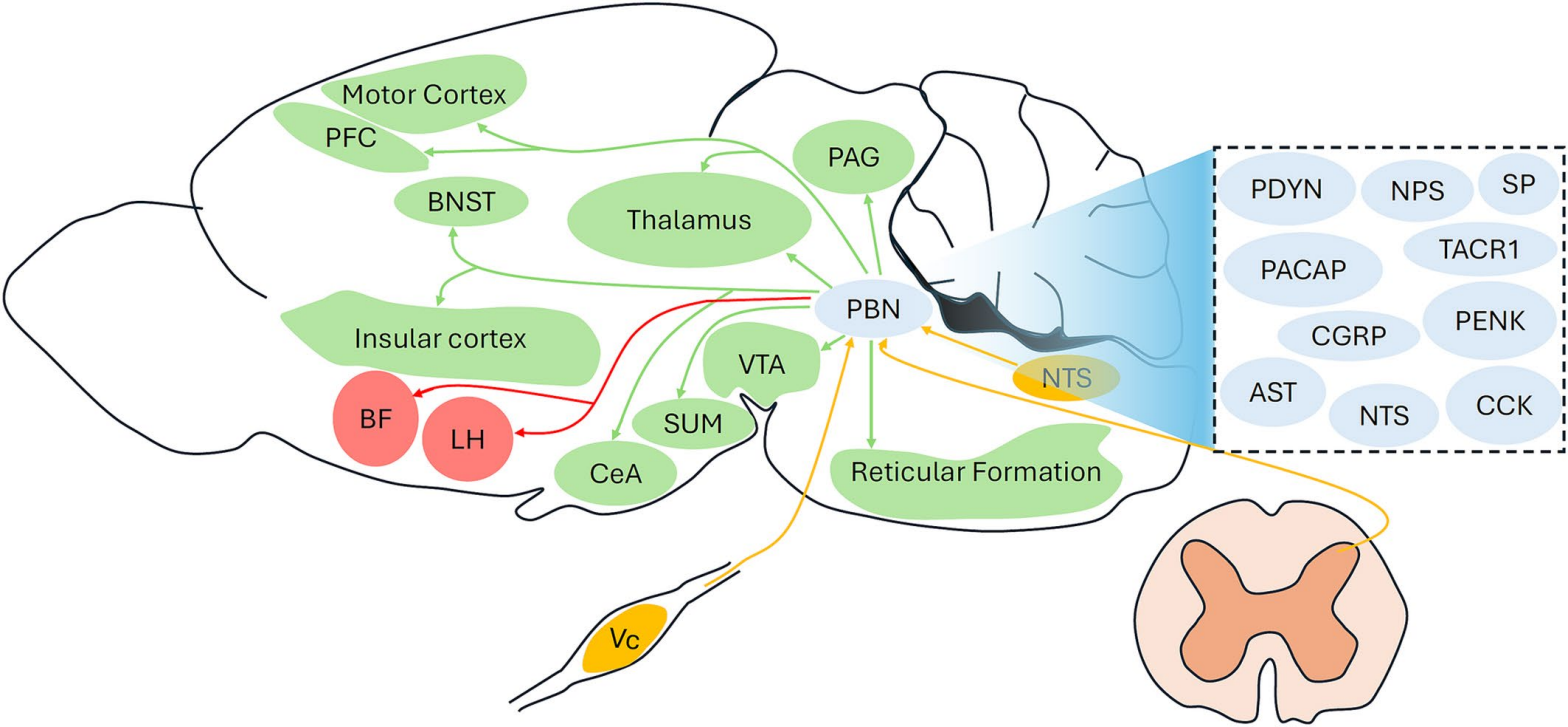
Identified and unidentified circuits involving the PBN under general anesthesia. Red arrows indicate identified PBN projections under general anesthesia; Green arrows indicate unidentified outputs from the PBN under general anesthesia; Yellow arrows indicate unidentified inputs to the PBN under general anesthesia. The dashed box includes the potential neuropeptides types of the PBN. PBN, parabrachial nucleus; PFC, prefrontal cortex; CGRP, calcitonin gene-related peptide; TACR1, tachykinin receptor 1; SP, substance P; CCK, cholecystokinin; NPS, neuropeptide S; PDYN, prodynorphin; PENK, proenkephalin; NTS, neurotensin; Vc, trigeminal spinal subnucleus caudalis; VTA, ventral tegmental area; PAG, periaqueductal gray; BNST, bed nucleus of the stria terminalis: CeA, central amygdala; LH, lateral hypothalamus; BF, basal forebrain; SUM, supramammillary nucleus.

The majority of neurons in the PBN are glutamatergic neurons, although there is a significant proportion of GABAergic neurons (Sun et al., 2020; Pauli et al., 2022). These two neuron types also coexpress several well-known peptides or receptors, such as calcitonin gene-related peptide (CGRP) (Palmiter, 2018), tachykinin receptor 1 (TACR1) (Deng et al., 2020), substance P (Barik et al., 2018), neurotensin (Chen et al., 2023), cholecystokinin (Yang et al., 2020), neuropeptide S (Xing et al., 2024), prodynorphin (Yang et al., 2020; Norris et al., 2021), and proenkephalin (Norris et al., 2021), and each population is associated with certain functions. The neuropeptide S- (Xing et al., 2024) and neurotensin-expressing neuron (Deng et al., 2020) in the PBN are associated with the regulation of sleep–wake behaviors. General anesthesia shares the classical feature of reversible loss of consciousness with natural sleep (Bao et al., 2023; Yang et al., 2022), and accumulating evidence has demonstrated that the activity of the PBN is also closely related to the actions of general anesthetics (Lu et al., 2023; Liu et al., 2023; Melonakos et al., 2021; Luo et al., 2018; Wang et al., 2019; Muindi et al., 2016). Here, we reviewed the current literature regarding the involvement of PBN in emergence from general anesthesia (Table 1). We also proposed unidentified PBN circuits related to general anesthesia (Figure 1).

**Table 1:** Study characteristics.

Study	Animals	Sex	Age	Anesthetics	PBN subregion	Main techniques	Cell type	Circuits	Effects of PBN activation on behavioral outcomes
Muindi et al. (2016)	Mice	Male	4–5 months	Isoflurane (1%)	Lateral	C-Fos staining; electrical stimulation; EEG; behavioral tests	NA	NA	Inducing RORR
Luo et al. (2018)	Rats	Male	Adult	Propofol (48 mg/kg/h); isoflurane (1.4%)	Nonspecific	C-Fos staining; fiber photometry; chemogenetic manipulation (DREADDs + CNO 5 mg/kg, intraperitoneally); EEG; behavioral tests	Neurons	PBN-PFC; PBN-BF; PBN-LH; PBN-TH; PBN-SUM	Shortening emergence time, without affecting induction time
Wang et al. (2019)	Mice	Male	8–10 weeks	Sevoflurane (2%)	Nonspecific	C-Fos staining; EEG; optogenetic and chemogenetic manipulation (DREADDs + CNO 1 mg/kg, intraperitoneally); behavioral tests	Glutamatergic neurons	PBN-PFC; PBN-MC; PBN-BF; PBN-LH	Shortening emergence time, prolonging induction time, increasing EC_50_ for LORR
Melonakos et al. (2021)	Rats	Male	Adult	Dexmedetomidine (0.3–4.5 μg/kg/min); ketamine (2–4 mg/kg/min)	Nonspecific	C-Fos staining; EEG; chemogenetic manipulation (DREADDs + CNO 1 mg/kg, intraperitoneally); behavioral tests	Glutamatergic neurons	NA	Prolonging emergence time under high-dose ketamine anesthesia
Liu et al. (2023)	Mice	NA	8–10 weeks	Isoflurane (1.4%)	Nonspecific	EEG; chemogenetic manipulation (DREADDs + CNO 3 mg/kg, intraperitoneally); behavioral tests	Astrocytes	NA	Shortening emergence time, increasing EC_50_ for LORR and RORR, without affecting induction time
Lu et al. (2023)	Mice	Male	8–10 weeks	Isoflurane (1.5%)	Nonspecific	C-Fos staining; optogenetic manipulation; EEG; behavioral tests	Glutamatergic neurons	PBN-LH; PBN-BF	Shortening emergence time, without affecting induction time

PBN, parabrachial nucleus; EEG, electroencephalography; PFC, prefrontal cortex; BF, basal forebrain; LH, lateral hypothalamus; TH, thalamus; SUM, supramammillary nucleus; MC, motor cortex; EC_50_, the concentration at which 50% of the mice lose or recover their righting reflex; LORR, loss of righting reflex; RORR, recovery of righting reflex; DREADDs, designer receptors exclusively activated by designer drugs; CNO, clozapine-*N*-oxide; NA, not available.

## Involvement of PBN in emergence from volatile anesthesia

### Isoflurane anesthesia

In 2016, Muindi et al. first reported the involvement of lateral PBN in emergence from isoflurane anesthesia (Muindi et al., 2016). Using c-Fos staining, they found that the activity of lateral PBN neurons increased during the period of emergence from 1% isoflurane anesthesia in mice. Notably, electrical stimulation of the PBN, but not the nearby region, induced cortical activation in the electroencephalogram (EEG), behavioral arousal and recovery of the righting reflex during isoflurane anesthesia, suggesting that the PBN serves as a wakefulness-promoting nucleus during isoflurane anesthesia. However, the cellular type of PBN that responds to isoflurane and whether stimulation with PBN affects isoflurane induction remain unclear. Lu et al. further investigated the involved PBN projections under isoflurane anesthesia (Lu et al., 2023). They showed that optogenetic activation of the PBN-lateral hypothalamus (LH) or PBN-basal forebrain (BF) projection induced cortical arousal on EEG in mice under 1.5% isoflurane anesthesia and promoted emergence from isoflurane anesthesia. Conversely, inhibition of these projections prolonged the emergence time. Interestingly, neither activation nor inhibition of these projections affected the induction time of isoflurane anesthesia. Regrettably, isoflurane sensitivity, i.e., the EC50 of the loss of righting reflex (LORR), was not examined to confirm the role of PBN projections in isoflurane induction.

Astrocytes in the central nervous system are involved in the regulation of sleep–wake behaviors (Chaturvedi et al., 2022; Bojarskaite et al., 2020). Liu et al. explored the contribution of astrocytes in the PBN to the loss of consciousness induced by isoflurane anesthesia in mice (Liu et al., 2023). They found that chemogenetic activation of PBN astrocytes induced cortical arousal (decreased delta power and burst suppression ratio) in EEG, increased the EC50 of loss of the LORR and recovery of the righting reflex (RORR), and shortened the emergence time from 1.4% isoflurane anesthesia in mice. However, the induction time was not affected. Conversely, chemogenetic inhibition of PBN astrocytes decreased the EC50 of the RORR and caused cortical inhibition (increased delta power and burst suppression ratio) on EEG. These findings indicate that PBN astrocytes play an important role in recovery from isoflurane anesthesia. Interestingly, although activation of the PBN-BF neural projection promoted the emergence from general anesthesia, direct activation of astrocytes in the BF facilitated isoflurane induction and delayed recovery time (Lin et al., 2023), suggesting that astrocytes in different arousal-related brain nuclei may have different roles in emergence from anesthesia. The contribution of astrocytes to general anesthesia may be attributed to the expression of many anesthetic targets, such as GABA_A_ receptors, glutamate receptors, sodium, potassium, and calcium ion channels (Mulkey et al., 2022; Chung et al., 2021). Previous studies have indicated that GABA_A_ receptors on astrocytes are targets for commonly used general anesthetics to induce cognitive dysfunction (Chung et al., 2021; Wang et al., 2024). However, the underlying molecular mechanism by which astrocytes modulate emergence from isoflurane anesthesia is not fully understood. It is generally thought that the effects of astrocytes on general anesthesia occur via interactions with neurons (Mulkey et al., 2022). However, several studies have indicated that general anesthetics suppress astrocyte calcium signals independently of neuronal activity, suggesting that the effects of general anesthesia on astrocytes may be generated via a nonneuronal mechanism (Thrane et al., 2012). Whether other glial cells, such as microglia and oligodendrocytes, regulate general anesthesia needs to be investigated because they also contribute to the regulation of sleep–wake cycles (Ma et al., 2024; Rojo et al., 2023).

### Sevoflurane anesthesia

The activity of the PBN is also involved in the general anesthesia induced by sevoflurane (Wang et al., 2019). Wang et al. showed that chemogenetic activation of PBN glutamatergic neurons shortened the emergence time of mice from 2% sevoflurane anesthesia, prolonged the induction time, and increased the EC50 of the LORR. Conversely, chemogenetic inhibition of PBN glutamatergic neurons produced the opposite effects. Optogenetic activation of PBN glutamatergic neurons induced cortical arousal according to EEG during the maintenance stage of sevoflurane anesthesia. They further showed that activation of PBN glutamatergic neurons induced excitation of several cortical and subcortical arousal regions, including the prefrontal cortex, motor cortex, basal forebrain, and lateral hypothalamus, under sevoflurane anesthesia. However, the specific role of these PBN projections under sevoflurane anesthesia has not yet been determined.

## Involvement of PBN in emergence from intravenous general anesthesia

In addition to volatile anesthesia, PBN activity also regulates consciousness under intravenous general anesthesia. Luo et al. used real-time calcium imaging to detect the activity of PBN neurons under general anesthesia induced by propofol and isoflurane (Luo et al., 2018). They found that the activity of PBN neurons was inhibited under propofol (48 mg/kg/h) and 1.4% isoflurane exposure but recovered during the emergence period in rats. The activation of PBN neurons induced cortical activation in EEG signals during the recovery period and shortened the emergence time of propofol and isoflurane anesthesia but had no effect on the induction time or on the EEG signals during the induction period. They further explored PBN projections and found that the prefrontal cortex, basal forebrain, lateral hypothalamus, thalamus, and supramammillary nucleus were activated when activating the PBN. However, the contributions of these PBN projections to general anesthesia were not further examined, nor did they identify the neuronal types of the PBN that respond to general anesthesia.

The modulation of the PBN by other types of intravenous general anesthetics, such as dexmedetomidine and ketamine, was different from that by propofol anesthesia. Melonakos et al. reported the role of PBN during dexmedetomidine and ketamine anesthesia in rats (Melonakos et al., 2021). Their data showed that activation of glutamatergic neurons in the PBN reduced delta power on EEG under low-dose (0.3 μg/kg/min) dexmedetomidine anesthesia but not under high-dose dexmedetomidine (4.5 μg/kg/min) or ketamine anesthesia (4 mg/kg/min), suggesting that these two drugs have distinct effects on delta power generation. They further found that activation of glutamatergic neurons in the PBN did not affect recovery time with dexmedetomidine anesthesia or with low-dose (2 mg/kg/min) ketamine anesthesia but did prolong recovery time with high-dose ketamine anesthesia. These findings suggest that PBN plays a different role in diverse groups of general anesthetics with different molecular targets and that other wake-promoting nuclei or circuits may play a more important role in the emergence of dexmedetomidine and ketamine anesthesia. However, they did not examine the effects of PBN activation on the induction process of dexmedetomidine and ketamine anesthesia. Moreover, additional experiments involving the inhibition of PBN neurons or optogenetic techniques will support these conclusions.

### Conclusion and perspectives

Recent studies have consistently revealed that activation of the PBN promotes recovery from general anesthesia induced by general anesthetics, including isoflurane (Lu et al., 2023; Liu et al., 2023), sevoflurane (Wang et al., 2019), and propofol (Luo et al., 2018), suggesting that the PBN plays a pivotal role in the recovery period of general anesthesia. Notably, activation of the PBN did not influence the recovery time of dexmedetomidine anesthesia or low-dose ketamine anesthesia but unexpectedly prolonged the recovery time of high-dose ketamine anesthesia (Melonakos et al., 2021). These findings suggest that the effects of PBN activation on general anesthesia depend on the molecular targets involved. In terms of anesthesia induction, there is still divergence. Manipulation of the activity of PBN did not affect the induction time of isoflurane (Lu et al., 2023) or propofol anesthesia (Luo et al., 2018) but did change anesthesia sensitivity, as evidenced by the altered EC50 of LORR (Liu et al., 2023). Only one study investigated the role of PBN in sevoflurane anesthesia (Wang et al., 2019). Interestingly, activation of PBN prolonged the induction time and increased the EC50 of the LORR under sevoflurane anesthesia (Wang et al., 2019). Future studies are still needed to confirm the involvement of PBN in anesthesia induction.

As the majority population of PBN neurons, glutamatergic neurons are identified as effective targets under general anesthesia (Lu et al., 2023; Wang et al., 2019). However, GABAergic neurons are assumed to constitute a definite proportion of PBN neurons ^29,^ and their contribution to general anesthesia needs to be investigated in future studies. Moreover, glutamatergic neurons can be further divided into several subpopulations, such as CGRP-expressing (Palmiter, 2018) and TACR1-expressing (Deng et al., 2020) neurons, and the specific role of these subpopulations under general anesthesia needs to be determined. The PBN projections involved in general anesthesia remain largely unknown because only the BF and LH have been identified as effective downstream targets of the PBN in isoflurane anesthesia (Lu et al., 2023). Although several regions, including the prefrontal cortex, thalamus, supramammillary nucleus, and motor cortex, are activated by PBN activation (Luo et al., 2018; Wang et al., 2019), whether these projections are associated with the actions of general anesthesia remains unclear. Additionally, it will be interesting to investigate whether other classical projections involving the PBN, such as the PBN-VTA (Zhang et al., 2021), PBN-BNST (Chiang et al., 2020), and PBN-CeA (Chiang et al., 2020), participate in the modulation of consciousness levels under general anesthesia. Figure 1 illustrates the identified PBN involving circuits (PBN-BF and PBN-LH) under general anesthesia and unidentified circuits, including common inputs and outputs of the PBN. Future studies are required to determine whether these circuits are associated with the actions of general anesthetics. As the PBN comprises different functional subregions (Chiang et al., 2019), its specific role in the regulation of general anesthesia should be examined. Notably, current evidence regarding the role of PBN in emergence from general anesthesia has mainly been obtained from male rodents, and whether there is a sex difference should be tested in future studies.

Despite the important involvement of PBN in the regulation of emergence from general anesthesia, limited research has investigated the underlying molecular mechanism by which general anesthetics influence the activities of PBN neurons or astrocytes. Ion channels or receptors distributed on PBN neurons or astrocytes may be molecular targets of general anesthetics. For example, recent studies have revealed that the sodium leak channel in glutamatergic neurons (Wu Y. et al., 2024) and Kir4.1 in astrocytes (Zhao et al., 2024) mediate the effects of sevoflurane on one wake-promoting region, the paraventricular thalamus. Additionally, a previous study showed that the sodium leak channel helps to maintain the neuronal activity of the PBN and regulates respiratory function under sevoflurane anesthesia (Wu L. et al., 2024). Therefore, it will be interesting to explore whether the sodium leak channel participates in the regulation of the emergence-promoting effects of PBN under general anesthesia. Considering that the expression of many peptides or receptors in PBN neurons, such as CGRP (Palmiter, 2018), TACR1 (Deng et al., 2020), substance P (Barik et al., 2018), neurotensin (Chen et al., 2023), cholecystokinin (Yang et al., 2020), neuropeptide S (Xing et al., 2024), prodynorphin (Yang et al., 2020; Norris et al., 2021), and proenkephalin (Norris et al., 2021), and several of them, such as CGRP (Kuroda et al., 2004) and TACR1 (Ishizaki et al., 1997), can be modulated by isoflurane or sevoflurane, future studies are required to determine whether these proteins are molecular targets that mediate the emergence-promoting effects of the PBN under general anesthesia. It is also meaningful to use *in-vitro* methods to investigate the direct effects of anesthetics on PBN neurons, which will provide the direct evidence for the involvement of PBN neurons in the general anesthesia.

One issue should be noted. Since PBN functions as a physiological relay nucleus for nociceptive information or warning signals (Torres-Rodriguez et al., 2024; Torruella-Suárez et al., 2024; Katagiri and Kato, 2020), which will also stimulate the brain’s arousal circuits and elevate arousal levels (Smith et al., 2023), thereby expediting recovery from general anesthesia. Therefore, sufficient arousal effects can be achieved by simply applying noxious stimuli without resorting to optogenetics, chemogenetics, or ultrasound stimulation methods. For example, the excitation of PBN neurons has already been reported to act as an unconditioned stimulus for fear conditioning and to alter pain sensitivity, leading to nociplastic pain (Palmiter, 2024), which will promote arousal. However, it is difficult to evaluate whether the observed phenomenon resulted from promoting emergence from anesthesia or merely from shifting the arousal-sedation balance. Both factors might contribute to this phenomenon, which is needed to be confirmed by future studies.

Accumulating animal evidence of the involvement of PBN in emergence from general anesthesia will facilitate the clinical translation in many conditions. For example, stimulating PBN may serve as a promising therapy to improve the patients’ recovery from general anesthesia or other unconsciousness conditions, such as coma. Moreover, selective manipulation of the neuronal subtype of PBN might provide more precise treatment and decrease the incidence of side effects. Recent evidence (Bian et al., 2021) indicated that noninvasive ultrasound stimulation of another emergence-promoting region, namely the ventral tegmental area, accelerated the emergence from isoflurane anesthesia in mice, highlighting the possibility of use noninvasive technique to stimulate PBN in clinic. It will be interesting if inhibition of PBN can be combined with the use of anesthetics to maintain the general anesthesia, which will reduce the consumption of general anesthetics as well as their related side effects, especially in patients with unstable respiratory and circulatory functions.
